# A case report of revision occipital-cervical fusion after atlanto-axial instrumentation failure for neurofibromatosis type I

**DOI:** 10.1186/s12893-019-0502-z

**Published:** 2019-04-25

**Authors:** Hayato Kinoshita, Naohisa Miyakoshi, Takashi Kobayashi, Toshiki Abe, Kazuma Kikuchi, Yoichi Shimada

**Affiliations:** 1 Akita Kosei Medical Center, 1-1-1 Nishibukuro Iijima, Akita, 011-0948 Japan; 2 Department of Orthopedic Surgery, Akita University Graduate School of Medicine, 1-1-1 Hondo, Akita, 010-8543 Japan

**Keywords:** Cervical spine, Neurofibromatosis type 1, Long fusion

## Abstract

**Background:**

Neurofibromatosis type 1 is an autosomal dominant genetic disease with characteristic café-au-lait spots, neurofibroma, and dystrophic changes in the bones. However, complications involving atlanto-axial dislocation are rare.

**Case presentation:**

We report a case of neurofibromatosis with atlanto-axial dislocation. The chief complaints were numbness of the upper limb and gait disturbance. We performed short fusion using the Brooks method. However, recurrence of the dislocation was found after 5 months recovery, and the patient underwent posterior fusion from the occipital bone to C4. Thereafter, she had a good postoperative course.

**Conclusions:**

Neurofibromatosis patients often exhibit a low bone mineral density because of dystrophic changes, and are prone to fragile bones. In the present case, the use of long fusion at the first surgery may have helped to form a strong fusion of fragile bone.

## Background

Neurofibromatosis type 1 (NF1) is an autosomal dominant disease, which is defined by the National Institute of Health as presenting with café-au-lait spots, neurofibroma, freckling of the axillary or inguinal region, glioma of the optic nerve, dystrophic bone disease, or family history [1]. The dystrophic changes of the bones involve rib penciling, scalloping of the vertebral body, costotransverse spindling, increased width between each pedicle, and expansion of the foramina. In a study of 102 patients with neurofibromatosis, patients with dystrophic changes (defined as dystrophic type) exhibited a rapid progression of spinal deformities, while conservative treatments were ineffective [2]. Kawabata et al. also reported an incidence of spinal deformity of 10–69% in neurofibromatosis patients [3], while Yong-Hing et al. reported cervical spine deformity in 17 of 56 neurofibromatosis patients (approximately 30%) [4]. However, the complication of atlanto-axial dislocation (AAD) is rare in cervical spine deformity, with only seven cases previously reported [5–9]. Herein, we report a case requiring two cervical spine surgeries because of recurring AAD.

## Case presentation

A 40-year-old woman consulted us with multiple café-au-lait spots, family history of neurofibromatosis, and prior diagnosis of NF1 by her primary doctor. Her chief complaints were numbness of the upper limb and gait disturbance from 1 month prior. Neurological examination revealed a spastic gait. The Romberg test was positive. The one leg standing test showed instability in both legs. Hyperreflexia showed a deep tendon of the biceps, triceps, patella, and Achilles on both sides. In the manual muscle test, only finger extension was reduced to 4 on the left hand. The sense of pain was reduced on the right side of her body.

**Fig. 1:**
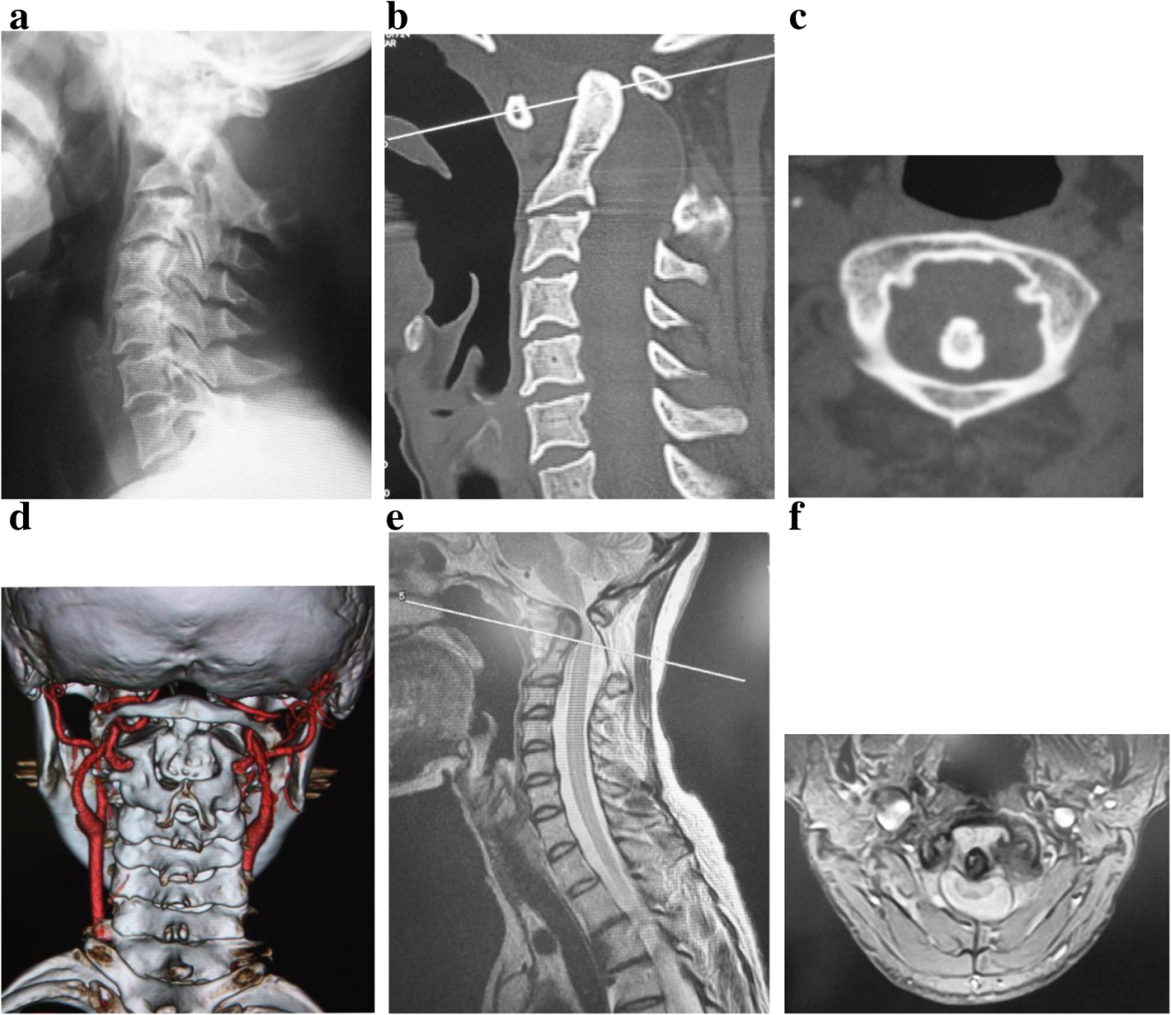
Preoperative imaging. **a** The cervical neural position showing expansion of the atlanto-dental interval. The upper atlantodental interval was 11.8 mm. **b** Computed tomography showing enlargement of the C2/3 foramen on the sagittal side of the left cervical spine. **c** Axial computed tomography image at the height of the C2 vertebra showing enlargement of the left side C2/3 foramen. **d** Computed tomographic angiography showing a left-side vertebral artery inside of the C1 lamina. **e** T2-weighted magnetic resonance imaging of the sagittal cervical spine showing spinal cord compression caused by a dislocated odontoid. **f** T2-weighted magnetic resonance cervical axial imaging showing disruption of the transverse ligament of the atlas and neurofibroma between the anterior arch of C1 and the odontoid

Radiography showed expansion of the atlanto-dental interval at the neutral position of the cervical spine (Fig. 1a), while canal stenosis was observed by computed tomography and magnetic resonance imaging (Fig. 1b, c, e). An abnormality of the left side vertebral artery inside of the C1 lamina was observed by computed tomographic angiography (Fig. 1d). Magnetic resonance imaging showed dural ectasia from C2 to T2, and AAD. There was no neurofibroma between the atlas and the odontoid (Fig. 1e, f).

**Fig. 2:**
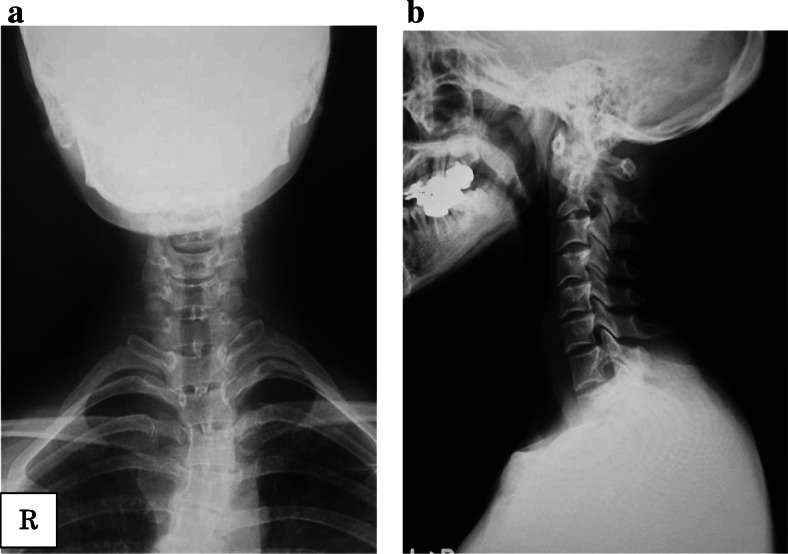
First operation. **a** Anterior–posterior radiography image at the first operation. **b** Sagittal radiography imaging at the first operation. The taping system and the iliac bone were used for fixation between C1 and C2. The upper atlantodental interval was 3.5 mm

**Fig. 3:**
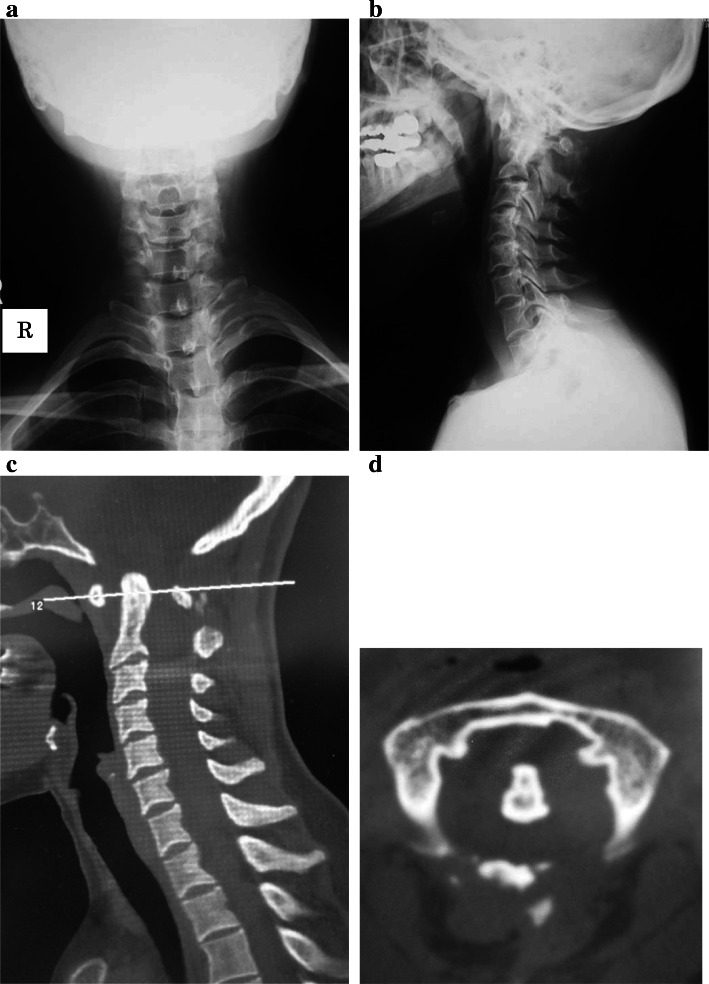
Recurrence of atlanto-axial dislocation. **a** Anterior–posterior radiography image. **b** Sagittal radiography imaging. The upper atlantodental interval was 8.5 mm. **c** Computed tomography sagittal image showing the recurrence of the atlanto-axial dislocation. **d** Computed tomography axial image showing the fracture of the C1 lamina

We performed surgery to prevent the progression of myelopathy caused by AAD. We initially planned a long posterior fixation. However, we achieved a good closed reduction of the AAD under general anesthesia. Thus, we tied an ultra-high molecular weight polyethylene cable (Nesplon; Alfresa, Inc., Osaka, Japan) to the C1 lamina and spinous process of C2 to maintain the position of the reduced AAD. Furthermore, we tied two nesplon cables® to the sublamina of C1 and C2 according to the Brooks technique. The iliac bone was grafted on between the C1 and C2 laminae (Fig. 2). The operative time was 1 h 35 min, and bleeding was < 50 ml. After the operation, the patient showed improvement of neurological symptoms. She wore a Philadelphia brace continuously. However, at 5 months after surgery she felt neck pain and consulted us again. Computed tomography showed fracture of the C1 lamina and recurrence of AAD (Fig. 3).

We reoperated using a long posterior fusion. Because of the existing abnormal vertebral artery inside of the C1 lamina, we gently removed scar tissue using a subperiosteal approach, and revealed the C1/2 facet under direct vision. We then introduced two intra-articular titanium spacers (KiSCO, Kobe, Japan) for fixed bilateral atlanto-axial joints.

**Fig. 4:**
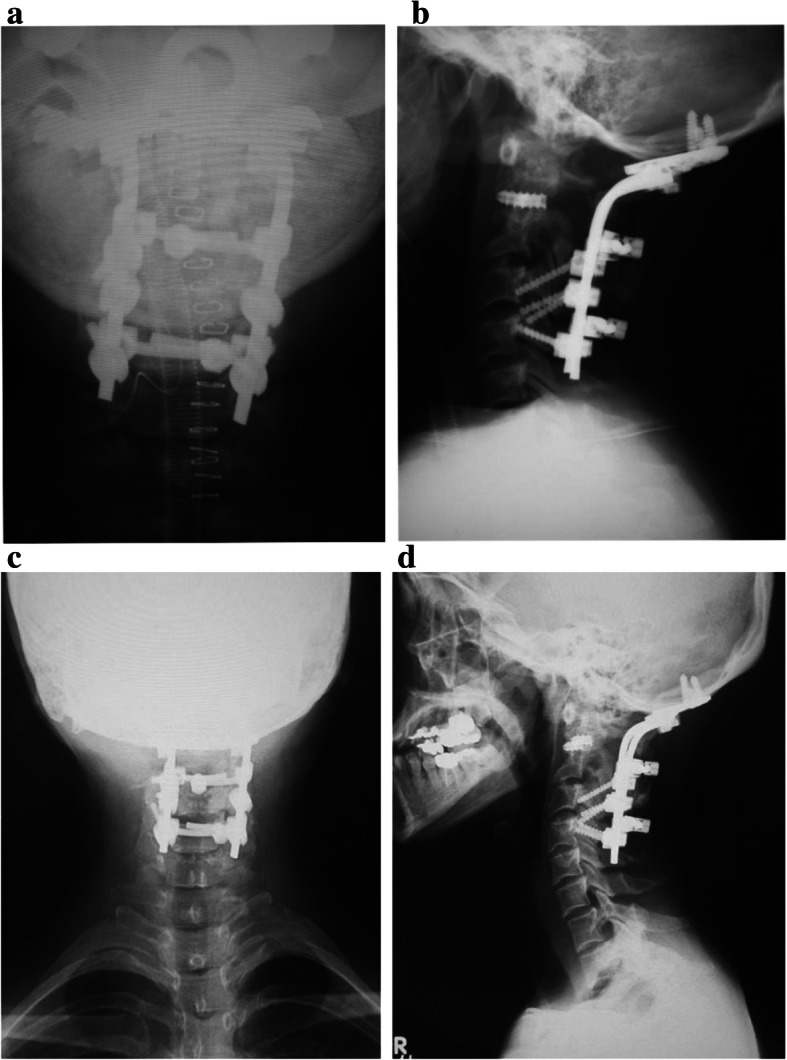
Second operation. **a** The anterior–posterior radiography image at the second operation. **b** Sagittal radiography image at the second operation. A long fixation from the occipital bone to C4 was performed. The upper atlantodental interval was 2.8 mm. **c** Anterior–posterior radiography image at 4 years after the second operation. **d** Sagittal radiography image at 4 years after the second operation

Vertex select® (Medtronic, Minneapolis, MN, USA), a plating system for occipital bone, was used for posterior fixation of the occipital bone and cervical spine. Facet screw fixations were inserted on the right side of C2/3 and both sides of C3/4. Lateral mass screw fixation was performed on both sides of C4. Two pre-bending rods were connected to these screws on both sides of the cervical spine, and two rod couplers were connected to the pre-bending rods at the height of C2 and C4. To avoid stress concentration and refractures, sublaminar taping was performed at C2, C3, and C4 using nesplon cables®. Finally, her right side iliac bone was grafted between the occipital bone and the back of the C1 lamina using the Newman technique (Fig. 4a, b). The operative time was 3 h 56 min, and bleeding was 425 ml. She wore a Philadelphia brace for 1 year after the second operation. At 4-year follow up, there was no AAD recurrence (Fig. 4c, d) and her neck pain had improved. She could walk independently, and a manual muscle test showed ‘normal’ for every muscle.

## Discussion and conclusions

AAD is a rare complication in neurofibromatosis patients. As a potential cause of AAD, Isu et al. reported that one of three neurofibromatosis patients with AAD had a neurofibroma between the anterior arch of C1 and the odontoid [7]. Craig et al. also reported one patient with neurofibromatosis requiring cervical spine surgery with AAD caused by disruption of the transverse ligament of atlas [8]. Furthermore, Hasegawa et al. suggested that AAD was caused by either abnormal development or neoplastic destruction of the joint [9]. In the present case, AAD was caused by disruption of the transverse ligament of atlas (Fig. 1f). In these four reported AAD cases, one case received laminectomy of C1, posterior fusion from the occipital bone to C3, and anterior fusion from C2 to C5; one case received posterior decompression of both the foramen magnum and C1, and posterior fusion from the occipital bone to C3; one case with neurofibromatosis between the odontoid and the anterior arch of C1 received removal of the anterior arch of atlas and odontoid displacement via a trans-oral approach, and C1–2 fusion; while one case used the Brooks technique [7, 8]. Importantly, all cases showed good outcomes. For postoperative treatment, external fixation was reported to maintain the correction and obtain solid bone union [10].

In the present case, we used the Brooks method, as previously reported [8], and used a neck collar for external fixation. We also used a nesplon cable® to fix the atlas and the axis. Fujita et al. showed that use of stainless steel wire or the nesplon cable® for sublaminar fixation provided equal stability [11]. Hamasaki et al. also reported that pedicle screw augmentation with a sublaminar nesplon cable® provided firmer fixation than pedicle screw augmentation only in the osteoporotic human thoracolumbar spine [12]. Furthermore, Saito et al. demonstrated no surgical complications such as lamina fracture in 44 patients with atlanto-axial subluxation who received the modified Brooks technique using ultra-high molecular weight polyethylene cable [13]. However, there are no reports on the use of nesplon cable® in NF1 patients. Of note, AAD recurred in our patient. Thus, further studies are required to confirm the utility of the nesplon cable® in NF1 patients with AAD.

Ito et al. compared the outcomes of 48 patients receiving posterior wiring using the McGraw or Brooks techniques with 28 patients receiving transarticular screw fixations using the Magarl technique [14], and found that nine of the 48 wiring patients did not obtain bone union, four of whom showed recurrence of atlantoaxial subluxation, while all patients receiving the Magarl technique obtained bone union. The authors suggested that even if posterior wiring was performed in AAD patients, sufficient postoperative rest periods and external fixation (e.g., Halo) would provide the best bone union and prevent recurrence of atlantoaxial subluxation. Our case wore a Philadelphia brace continuously after the first operation, but AAD recurrence occurred. Thus, it may have been useful to perform transarticular screw fixation (e.g., using the Magarl technique) at the first surgery, taking care to look for abnormal vertebral arteries. It is also important to note that there are potential disadvantages when using cables for C1–2 fixation. For example, Yoshimoto et al. reported that C1–2 fixation in a hyperlordotic position caused subaxial kyphosis after surgery, and progression of kyphosis occasionally caused additional myelopathy [15].

In the present case, we performed a second surgery using intra-articular spacers with a long fixation from the occipital bone to C4. Tokuhashi et al. reported the efficacy of C1–2 intra-articular screw fixation for atlanto-axial subluxation, including no changes in the atlas-dens interval during follow-up, no impairment of walking ability, and no surgery-related complications in rheumatoid arthritis patients [16]. Similarly, our case showed improvement in preoperative symptoms and no AAD recurrence. We suggest that the secondary AAD in our case was caused by fracture of the C1 lamina (Fig. 3), rather than rupture of the C1–2 fixation site. The only obvious dystrophic change in our case was expansion of the left C2/3 foramina, with no evidence of other dystrophic changes such as rib penciling, scalloping of the vertebral body, costotransverse spindling, or increased width between each pedicle. Furthermore, there was no evidence of the rapid progressive spinal deformities reported in dystrophic type neurofibromatosis [2]. Thus, we suggest that the fracture of the C1 lamina in our case was not caused by rapid spinal deformity. Stevenson et al. reported decreased bone mineral density in children and adolescents with NF1, with or without skeletal abnormalities, compared with controls [17]. Lammert et al. also reported a marked decrease in bone mineral density of NF1 patients who required surgical treatment of scoliosis [18]. Based on these reports, the selection of a long rigid fixation because of potential bone fragility may have prevented the secondary AAD in our case.

## Abbreviations

AAD: Atlanto-axial dislocation
NF1: Neurofibromatosis type 1
